# Report of the Diseases of Edinburgh for May, 1809

**Published:** 1809-07

**Authors:** John Roberton


*Report of the Diseases of Edinburgh for May*, 1809.

By JOHN ROBERTON, M. D.

The weather, in the beginning of this month, was soft, and accompanied by occasional gales of wind from the west. In this state, or nearly so, it continued till about the end of the first third of the month, when, although cloudy, it was very good, and afforded some warm and very pleasant days. About the beginning of the last third, the easterly winds prevailed and fogs became common, particularly early in the morning and after sunset. This sort of weather was, in a few days, succeeded by showers of rain, and we were assailed, within the last few days of the end of the month, with the coarsest weather that has been witnessed in this part of the world for many years. Hail, rain, and snow, fell in amazing quantity in the neighbourhood of Edinburgh for a whole day and night, causing the surrounding hills to exhibit rather the appearance of the month of January than of the end of May: the weather too was excessively cold. On the following day, a similar fall took place in the city of Edinburgh, and the flakes of snow have not been surpassed in size, at least within my remembrance, even in the depth of winter. The weather at the termination of the month was soft.

The barometer, about the beginning of the month, fell considerably. About the end of the first third, however, it rose a little, and continued to vary till near the end of the month, when, during the storm, it again fell.

The thermometer, till near the end of the month, stood between 45 and 60; but, toward the end of the month, it fell considerably below this.

The complaints which have been most prevalent have been pneumonia, enteritis, chin-cough, measles, small-pox, and fever.

The cases of pneumonia which have occurred in practice have been exceedingly bad. The disease, however, has not been nearly so frequent in occurrence as usual for several months past, but it has often terminated fatally in the space of a few hours. From our habit of annually witnessing the regular recurrence of inflammatory complaints during the winter months, the generality of people here seldom, at that time, attempt to account for their existence; but the usual season of such affections having elapsed, and their severity seemingly in a great measure having abated, their recurrence, especially to such a pitch of severity as has lately happened, excited the wonder and astonishment astonishment of every one. The perpetual anxiety in the human mind rather to give a ridiculous explanation of an event than no explanation at all, was clearly evinced on the present occasion. The water by which the city is supplied, was fixed on as the criminal on this occasion, and no sooner was this report put into circulation, than every one could easily point out the very cause of these complaints actually floating in the fluid. The majority seemed to think that it was owing to some insect which had found its way into the water in immense numbers, and, so convinced were they of the fact, that, in almost every glass of water, they could point out some of them. So they might have done last year, or any other year at the same season, for they were literally the small harmless summer insects, which, unfortunately for themselves only, are at this season usually drowned in the water. These complaints, however, before the end of the month, had almost all disappeared; but, in some enfeebled patients, there remained after them a sort of chronic cough, which I found uniformly removable by the administration of pills, composed, as I formerly mentioned, of myrrh, ammoniac, squills, and a small proportion of opium. During the violence of the pneumonia, the most active practice was found absolutely necessary. Large and repeated bleeding, blisters applied over the whole chest, with the administration of saline cathartics, were often all found necessary at the same time. I am sorry to say that even the most active practice was frequently found ineffectual, and consequently many patients died.

Enteritis, although severe, was by no means so much so, or so fatal as the last mentioned complaint. Some patients died of it, but the generality recovered by the judicious abstraction of blood, and by the application of blisters over the abdomen.

Chin-cough has occurred in various parts of the city, but it never has been so severe as to excite much general alarm. No instance that has come within my knowledge, or under my immediate observation, has terminated fatally. Measles and small-pox have also had their place in the list of diseases; but neither their extent nor severity has been of an alarming nature. One or two cases of small-pox seemed rather of a bad kind, but the patients recovered.

Fevers, as usual, still continue to prevail in this place, and also, as usual, are found to originate and prevail with greatest virulence in the nasty abodes of the poor.

*No. 12, Prince's Street.*

